# Egg burying behaviour in *Pristimantis* highlights the link between direct development and specialised parental care

**DOI:** 10.1002/ece3.10808

**Published:** 2023-12-13

**Authors:** Francesca Nicole Angiolani‐Larrea, Lelis Jindiachi, José Gabriel Tinajero‐Romero, Anyelet Valencia‐Aguilar, Marina Garrido‐Priego, Jaime Culebras, Eva Ringler

**Affiliations:** ^1^ Division of Behavioural Ecology, Institute of Ecology and Evolution University of Bern Bern Switzerland; ^2^ Pueblo Shuar Arutam Federacion Interprovincial Centros Shuar (FICSH) Sucúa Ecuador; ^3^ Photo Wildlife Tours Quito Ecuador; ^4^ Fundación Cóndor Andino Quito Ecuador; ^5^ Present address: Escuela de Biologia Universidad de Costa Rica San José Costa Rica

**Keywords:** Anura, oviposition, parental care, *Pristimantis chocoensis*, reproductive strategies, terrestrial eggs

## Abstract

One of the most extreme adaptations to terrestriality in anurans is direct development, where eggs from terrestrial clutches entirely circumvent an aquatic tadpole stage and directly develop into small froglets. We here report the first case of egg‐burying behaviour in a neotropical direct‐developing frog with subsequent short‐term maternal care. An amplectant pair of *Pristimantis chocoensis* was found at the Reserva Canandé in Esmeraldas, Ecuador, and we recorded oviposition and the later rotation and active burying of the clutch by the female. Both parents remained close to the nest area the following day. This rare observation sheds light on the complex but often cryptic reproductive behaviours of terrestrial amphibians and suggests that the evolution of direct development has selected for highly specialized forms of parental care.

## Graphical abstract

We report the first case of egg‐burying behaviour in a neotropical direct‐developing frog. Both parents remained near the nest during the following day. This rare observation sheds light over the intricate reproductive behaviours of terrestrial amphibians and suggests that the evolution of direct development has selected for highly specialized forms of parenting.

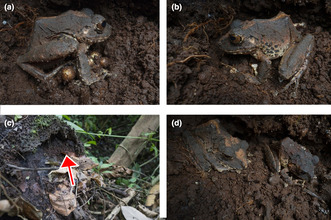

## Resumen

Una de las adaptaciones más extremas a la terrestreidad en los anuros es el desarrollo directo, donde los huevos de puestas terrestres evitan por completo la etapa de renacuajo acuático y se desarrollan directamente en versiones miniatura de los adultos. Aquí informamos el primer caso de comportamiento de enterrar huevos en una rana neotropical de desarrollo directo con posterior cuidado materno a corto plazo. Una pareja amplectante de *Pristimantis chocoensis* fue encontrada en la Reserva Canandé en Esmeraldas‐Ecuador, y registramos la oviposición y la posterior rotación y enterramiento activo de la puesta por parte de la hembra. Ambos padres permanecieron cerca del área del nido durante el día siguiente. Esta rara observación arroja luz sobre los complejos, pero a menudo crípticos, comportamientos reproductivos de los anfibios terrestres y sugiere que la evolución del desarrollo directo ha seleccionado formas altamente especializadas de cuidado parental.

## 1 BACKGROUND

Many animal species invest in the construction of breeding sites to improve the environmental conditions experienced by their offspring (Hansell, 2005; Mainwaring et al., 2017). For example, several species build nests or dig burrows to deposit their offspring in specific locations. This provides additional protection from environmental threats and predators and, as a consequence, increases the offspring's chances of survival (Mainwaring et al., 2017; Smiseth et al., 2012). In amphibians, nest building is largely associated with the evolution of terrestrial reproduction in order to keep eggs safe and hydrated (Wells, 2007). For example, in some anuran species, parents build (semi‐)terrestrial foam nests composed of aerated secretions, which provide shelter for eggs and sometimes for tadpole development (Gould, 2021). These nests may provide a buffer against thermal extremes, desiccation and predators (Gould et al., 2020), or improve access to oxygen and nutrients stored in the froth itself (Gould, 2021). Other anuran species also build nests using elements found in their habitat. For example, in gladiator frogs (*Boana faber*), males modify the structure of the mud in the margins of ponds to provide stable conditions for eggs to develop. These structures provide protection even to the parents themselves (Luza et al., 2015). All these diverse and complex parental strategies are thought to have facilitated the transition from aquatic to terrestrial reproduction in amphibians (Ringler et al., 2023; Vági et al., 2019).

One of the most extreme adaptations to terrestrial reproduction in anurans is direct development, where eggs from terrestrial clutches entirely circumvent an aquatic tadpole stage and directly develop into small froglets. This reproductive mode has evolved several times independently across different anuran, urodelan and gymnophionan lineages (Callery et al., 2001; Furness et al., 2022; Gomez‐Mestre et al., 2012; Liedtke et al., 2022). In Neotropical anurans, it is found in several species across the families Strabomantidae, Eleutherodactylidae, Craugastoridae, Bufonidae, Brachycephalidae and Hemiphractidae (Callery et al., 2001; Castroviejo‐Fisher et al., 2015; Liedtke et al., 2022; Romero‐Carvajal et al., 2023; Townsend & Stewart, 1985). Direct development is considered the most extreme adaptation to terrestrial life in amphibians, as it allows species to become completely independent from water bodies for reproduction (Gomez‐Mestre et al., 2012; Wells, 2007).

Frogs of the genus *Pristimantis* (Strabomantidae) Jiménez de la Espada, 1870, occur in north‐western South America and currently form the most speciose vertebrate genus in the world, with 605 described species (Frost, 2023). It is also the genus with the highest number of endemic species in Ecuador (Ortega‐Andrade et al., 2021). Currently, there is limited knowledge about the reproductive behaviour of *Pristimantis* frogs, particularly with respect to parental care. Here, we report the first observation of egg‐burying behaviour in the genus *Pristimantis*, which is followed by short‐term egg guarding by the female.

## 2 FIELD OBSERVATIONS

**FIGURE 1:**
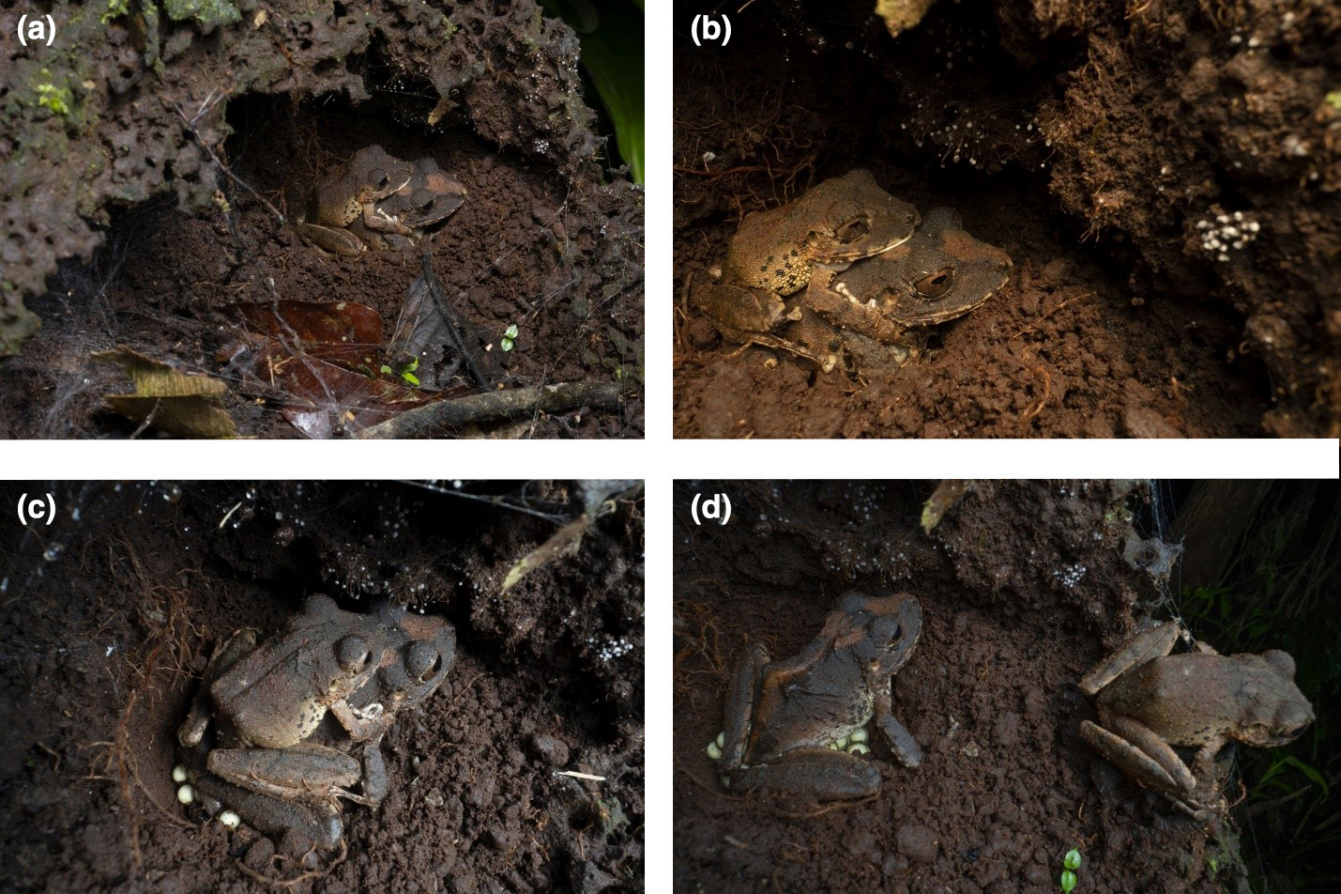
Amplexus and oviposition of *Pristimantis chocoensis* in Reserva Canandé‐Ecuador. (a) and (b) show both (male and female) adult individuals in the amplexus; (c) shows a snapshot of the oviposition; and (d) shows the male close to the female right after oviposition.

We found a *Pristimantis chocoensis* (Reyes‐Puig et al., 2020) couple in amplexus on June 3, 2022, at 02:16 AM inside an abandoned termite nest attached to a fallen trunk (Figure 1a). It was located right next to a trail and a steep slope of 2–3 m within the Río Canandé Reserve, in the province of Esmeraldas, Ecuador (0.52510, −79.21150 at 350 m.a.s.l.). The termite nest showed signs of degradation; it had a big opening in the upper side and loose soil inside the nest. The amplectant pair remained in the same spot and position for nearly 2 h in what appeared to be a depression in the soil. We cannot confirm that this depression was a hole dug by any of the adults or if it was already part of the topography of the termite's nest. At 4:02 AM, both egg and sperm release were detected (Figure 1b). After a few minutes, the female closed its eyes, raised its snout and stretched its body slightly, which we suspect was accompanied by the release of a second batch of eggs. At the same time, the male showed abdominal contractions, which we interpreted as the release of sperm for egg fertilization. After egg release had been completed, the male remained on the female's back for a period of 4 min, during which it exhibited additional abdominal contractions, which very likely released more sperm. The male subsequently detached from the female and moved a few centimetres away (Figure 1d).

**FIGURE 2:**
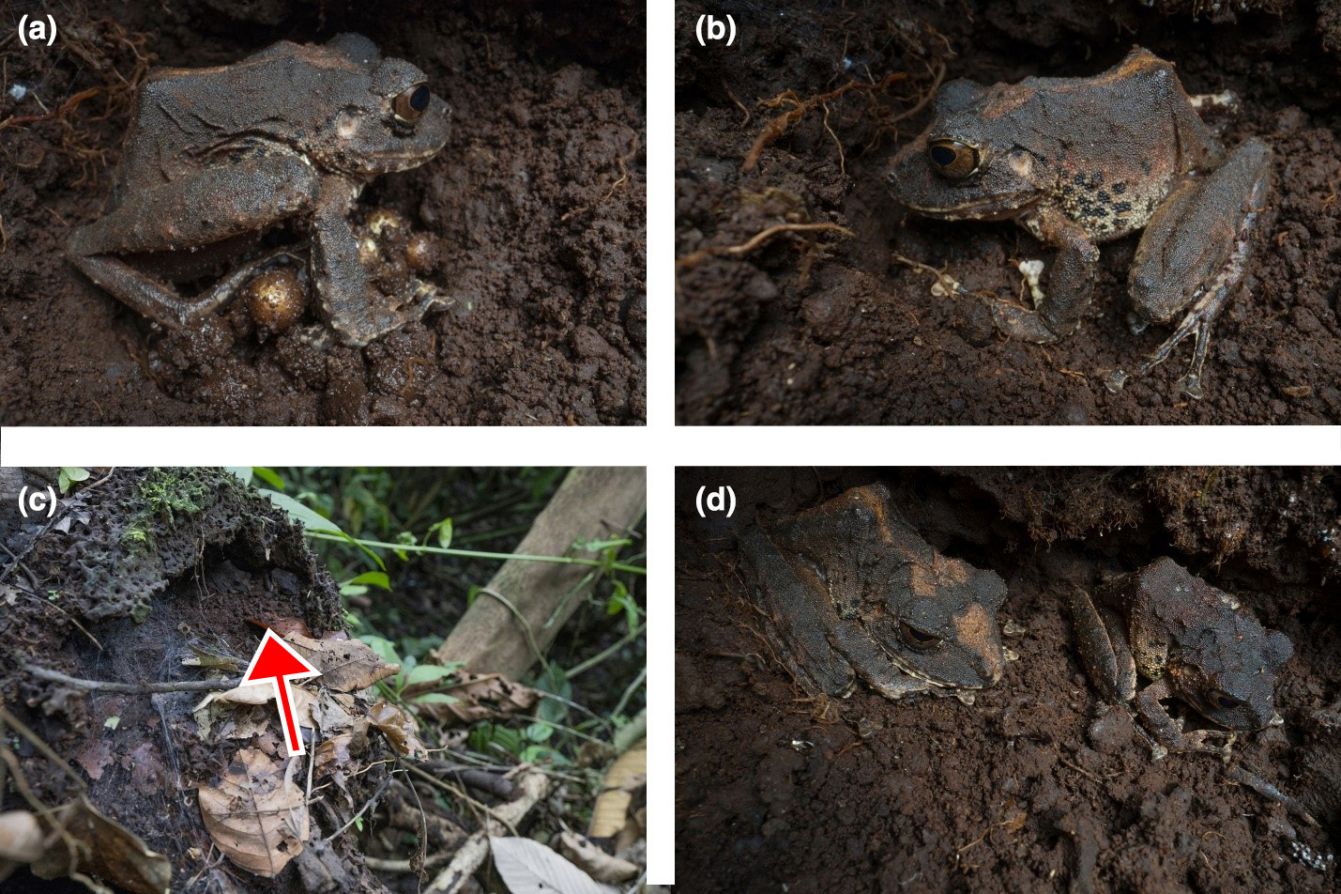
Post‐oviposition behaviour and oviposition site of *Pristimantis chocoensis* in Reserva Canandé‐Ecuador. (a) Female rolling the eggs after oviposition; (b) eggs almost completely buried; (c) and (d) male and female together in a nest during the day time, eggs completely buried underneath the female. Red arrow showing placement of adult individuals.

The female released at least 20 eggs and remained on top of them, changing positions of its body clock‐wise and counter‐clock‐wise every few minutes for approximately 1 h, rotating the eggs with its hind legs (Figure 2a). Rotation was performed by scrambling movements of the hind legs, one leg at a time, and thereby the eggs got mixed and covered with soil. At 05:14 AM, the female was observed scooping up soil from the surrounding area by pulling the loose soil underneath its body towards the eggs with its hind legs until the eggs were completely buried, forming a mound (Figure 2b). During this entire process, the female kept the eggs underneath its body. This behaviour continued at least until 06:10 AM, but the exact time at which it stopped was not recorded. The male remained close to the female at the border of the termite nest, immobile and not calling throughout the entire process.

The oviposition site was checked later that same day at 03:00 PM. The adult male and female were found in a resting position, in similar locations as they were 9 h earlier, the female on top of the mound and the male a few cm away from the female. Both frogs had their front and hind limbs pushed close to the body, their snout and ventral area flattened to the ground, and their eyes closed, they were seemingly sleeping (Figure 2c,d). On the second night of observation, the male was no longer at the oviposition site or visible in the immediate surroundings, but the female was still sitting on top of the mound formed over the buried eggs (Table 1; Video 1). Neither the female nor the male were observed near their nest on the following seven nights.

**TABLE 1:** Times of observed breeding behaviours in *Pristimantis chocoensis* at Reserva Canandé.

Date	Time	Observed behaviour
3 June	02:16 AM	Individuals found in amplexus
3 June	04:02 AM	Oviposition occurs and the male releases sperm
3 June	04:09 AM	Female releases second batch of eggs and male releases more sperm
3 June	04:13 AM	Amplexus stops, but male keeps on the back of the female
3 June	04:17 AM	The male possibly releases more sperm before moving away from the female. The female begins to rotate the eggs
3 June	05:14 AM	The female begins to bury the eggs in soil, producing a mound
3 June	06:10 AM	The female continues burying the eggs
3 June	02:56 PM	The female and the male remain at the site along with the buried eggs
3 June	11:06 PM	The female is observed sitting on top of the buried eggs. The male has left the site
4 June	08:00 PM	No parental care is observed

**VIDEO 1:** Summary of the reproductive activity of *Pristimantis chocoensis* in Reserva Canandé‐Ecuador. After amplexus and oviposition (00:20), the female is seen rotating the eggs (00:50) and subsequently covering the egg mass with soil (01:23). Finally, females and males are seen seemingly sleeping at the oviposition site (01:51) in the morning after oviposition activity, and the female was observed at the same location after approximately 24 h (02:10). Observations started at 02:16 AM and were recorded under red light conditions to avoid any disturbance to the individuals. Only once (minute 01:06) we shortly switched to white light, to obtain better visibility of the female's behaviour and the surrounding area.

## 3 DISCUSSION

In this report, we present the first case of egg‐burying behaviour combined with short‐term egg guarding in the genus *Pristimantis*, an extremely rare and specialized behaviour in anurans. Amphibians exhibit a great diversity in reproductive modes, many of which include some form of parental care. For species with direct development, the following reproductive modes, which include some form of parental care, have been described: guarding of terrestrial eggs; transportation of freshly hatched froglets by either males or females; brooding of eggs in dorsal pouches or basins; brooding of eggs embedded in dorsum of aquatic species; brooding of eggs in the dorsum depressions where froglets have specialized gills; and covering of eggs with dirt (Schulte et al., 2020). None of these modes include active burying of eggs plus egg guarding, as described herein (Liedtke et al., 2022; Schulte et al., 2020). Species with direct development are particularly interesting to better understand the adaptations for the evolutionary transition from aquatic to terrestrial environments for reproduction in amphibians. Our report thus highlights the importance of natural history information for species with highly cryptic behaviours.

In this report, we describe the short parental care that occurs in *P. chocoensis*, in which the female rolls and buries the eggs and finally covers the clutch with dirt. Similar behaviour has been reported in other anuran genera with direct development. Some species of *Philautus* are known to dig a nest prior to oviposition, then rotate their eggs and cover them with a thin layer of soil (Bahir et al., 2005). Also, in the pumpkin toadlet, *Brachycephalus ephippium*, females lay their eggs in the ground and cover them with soil (Pombal et al., 1994). As we detected the *P. chocoensis* pair after amplexus had already commenced, it is difficult to determine whether they initially dug a hole in the ground prior to egg release. However, the adult pair was located in a noticeable depression in the ground, which was subsequently used for egg deposition. Furthermore, the male and female's feet and legs were covered in soil, which suggests that there might have been active digging by at least one of the adults prior to oviposition.

Similar to other direct‐developing and nest‐building species (e.g. Bahir et al., 2005; Pombal et al., 1994), we observed in *P. chocoensis* egg rotation prior to burying. This behaviour has been proposed to provide various advantages to the developing eggs. The layer of soil around the eggs may provide camouflage (Pombal et al., 1994), and with an extra soil layer as cover, eggs are also protected from UV‐B radiation exposure. This has been shown to negatively impact egg development if over a critical threshold (Ovaska et al., 1997; Palen et al., 2005). However, egg burying in *P. chocoensis* already serves to hide from predators and protect against UV radiation exposure; hence, we believe that the most plausible explanation for the egg rotation behaviour is to spread sperm more evenly across the eggs to enhance fertilization success (cf. Delia et al., 2017). Further research is needed to identify the adaptive significance of egg rolling behaviour in this species and other terrestrial breeding anurans.

Our observations show that both adults remained either near or directly on top of the buried eggs for several hours (female: approx. 24 h; male: approx. 12 h) after oviposition. This is in contrast to what has been reported from *Philautus* species and *B. ephippium*, in which males typically abandon the oviposition site immediately after fertilization and females abandon the site after nest building is completed. As we do not have additional information about the species' reproductive behaviour, we cannot unambiguously confirm whether the presence of the male was intended guarding behaviour or if it was coincidental. Further research is needed to better understand the functions of prolonged male presence at the oviposition site in *Pristimantis* frogs. For the female, we interpret the remaining at the clutch site over 24 h as active egg guarding behaviour.

In other species with direct development that do not bury their eggs, egg guarding is much more prolonged (i.e. several days or weeks) as eggs are more exposed to eventual predators, and extended guarding seems to be critical for clutch survival. In contrast to that, we interpret the behaviour of *P. chocoensis* as a result of a shift in energy allocation from low‐intensity long‐term care (i.e. egg guarding) to high‐intensity short‐term care (i.e. egg burying and very short‐term egg guarding).

Previous research suggested that direct development is already a derived and complex adaptation to terrestrial reproduction, and therefore selection for additional forms of parental care is low (Vági & Székely, 2023). However, increasing numbers of reports from species that exhibit direct development and also provide parental care challenge this hypothesis (Furness & Capellini, 2022; Schulte et al., 2020). Besides nest‐building activities, there are species with direct development where either the female (e.g. Chinchilla‐Lemus & Meneses‐Pelayo, 2016; Mebert et al., 2022; Ortega‐Andrade et al., 2010; Pombal et al., 1994) or the male (e.g. Bignotte‐Giró et al., 2022; Chinchilla‐Lemus & Meneses‐Pelayo, 2016; Mebert et al., 2022; Rojas‐Rivera et al., 2013) care for the recently laid eggs, and in some cases, parents of these species carry newly hatched froglets over several weeks on their backs to provide protection (Diesel et al., 1995; Schulte et al., 2020). All these examples, including the observation presented in the present report, suggest that highly specialized forms of parental care may be adaptive in direct developing species. For example, the burying of eggs in soil without access to water can mainly become adaptive in direct‐developing species, as most other amphibians have larvae with an obligatory aquatic phase.

Amphibians possess an immense diversity of reproductive modes, yet for a large number of species, detailed life history information is missing (Schulte et al., 2020). Natural history observations are especially valuable for species that are very rare or feature behaviours that are very difficult to observe. Only with detailed natural history knowledge we will be able to understand the causes and consequences of variation in reproductive behaviours, and the interaction between ecology, physiology and behaviour in the evolution of parental care.

## AUTHOR CONTRIBUTIONS

**Francesca Nicole Angiolani‐Larrea:** Conceptualization (equal); data curation (supporting); formal analysis (equal); investigation (supporting); validation (equal); visualization (equal); writing – original draft (lead); writing – review and editing (equal). **Lelis Jindiachi:** Investigation (supporting); validation (supporting); writing – original draft (supporting). **José Gabriel Tinajero‐Romero:** Investigation (supporting); validation (supporting); writing – original draft (supporting). **Anyelet Valencia‐Aguilar:** Validation (supporting); writing – review and editing (equal). **Marina Garrido‐Priego:** Validation (supporting); writing – review and editing (equal). **Jaime Culebras:** Conceptualization (equal); data curation (equal); formal analysis (equal); investigation (equal); supervision (equal); validation (equal); visualization (equal); writing – original draft (supporting); writing – review and editing (equal). **Eva Ringler:** Conceptualization (equal); funding acquisition (lead); project administration (lead); supervision (equal); validation (supporting); writing – review and editing (equal).

## CONFLICT OF INTEREST STATEMENT

The authors disclose no conflict of interest.

## Data Availability

Data sharing not applicable to this article as no datasets were generated or analysed during the current study.
